# Scalable CO_2_ Removal Using Electricity: Research Needs in Bipolar Membrane Electrodialysis

**DOI:** 10.1021/acsenergylett.5c01927

**Published:** 2025-09-02

**Authors:** Zarko P. Jovanov, Dingchang Yang, Carla Glassl, Qilei Song, Ifan E. L. Stephens

**Affiliations:** † Ucaneo Biotech GmbH, c/o Berlin Industrial Group, Schwarze-Pumpe-Weg 16, 12681 Berlin, Germany; ‡ 4615 Imperial College London, Department of Chemical Engineering, South Kensington Campus, SW7 2AZ London, United Kingdom; § 4615 Imperial College London, Department of Materials, South Kensington Campus, SW7 2AZ London, United Kingdom

## Abstract

Electrochemical direct air capture (DAC) potentially represents a transformative solution in the fight against climate change. Herein we provide a critical perspective on pH-swing approaches, which leverage pH shifts to enhance CO_2_ capture and release while minimizing energy demands and improving scalability. In particular, bipolar membrane electrodialysis offers promise. Widespread adoption requires overcoming challenges across molecular, micro-, and system scales. At the molecular scale, development of durable ion exchange layers and their integration into bipolar membranes with high ion conductivity, permselectivity, and stability is critical. At the microscale, stack designs must minimize energy losses by optimizing fluid dynamics, current densities, faradaic efficiencies, and pH gradients. At the system level, scalable DAC systems must integrate renewable energy and advanced CO_2_ uptake strategies, such as enhanced solvent/electrolyte formulations and innovative reactor configurations. By addressing these milestones, electrochemical DAC can achieve intensified processes with improved energy efficiency, reduced costs, and higher CO_2_-removal capacities.

## Graphical abstract

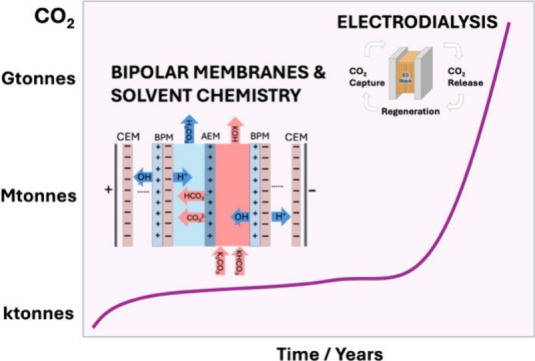

Carbon dioxide removal (CDR) is critical in addressing climate change and achieving global decarbonization targets. The Intergovernmental Panel on Climate Change (IPCC^1^) highlights that CDR is essential to counterbalance hard-to-abate residual emissions and is a key component in scenarios limiting global temperature rise to 1.5 or 2.0 °C. Hybrid methods for CDR,^1–5^ such as enhanced rock weathering, or natural as use of plants and the large-scale reforestation can help offset emissions, where as permanent, durable and large-scale capture (>100 Mtonnes CO_2_/year) and sequestration of CO_2_ at higher rates require DAC^6–17^ or direct ocean capture^18–21^ technologies. Currently deployed heat-based DAC systems take advantage of low energy costs at centralized locations endowed with waste heat^22^ or geothermal heat sources.^23–28^ In comparison, electrochemical approaches are more amenable to decentralized DAC as they can be solely run on renewable electricity, and can be integrated with intermittent sources of power, such as wind and solar.^26,29^ However, the current cost of grid electricity at 170–530 €/MWh is too expensive.^30,31^ Moreover, the need for specialized materials^11,32^ and equipment imposes high capital costs.^33^

In this Perspective, we feature the challenges and opportunities of bipolar membrane electrodialysis (BPM-ED) for solvent regeneration and CO_2_ release in DAC.^21,34–43^ We relate the performance of selected simple electrochemical systems and devices using functional membranes and catalysts as the key components. Most importantly, we examine the factors influencing their energy efficiencies and emphasize the importance of the overall process intensity for long-term cost structure. We highlight the most relevant scientific and engineering research topics promising further cost reductions.

In broad terms, DAC involves two primary processes: CO_2_ uptake (absorption^44^ or adsorption) and CO_2_ release (recovery).^39,40,45,46^ In the CO_2_ uptake process, through aeration, sorbent or solvent material is brought into contact with ambient air with a low CO_2_ content of approximately 450 ppm. CO_2_ molecules diffuse from the air into the sorbent, solid material or liquid solvent, eventually reaching a maximum concentration of *dissolved inorganic carbon* (DIC) at thermodynamic equilibrium.^47,48^ During the release process, gas phase CO_2_ is recovered and released at higher concentrations and higher purity, suitable for further utilization^24,49,50^ or storage.

In Figure 1a, we show how in a pH swing process, an electrochemical device, such as a BPM-ED unit, links the two major processes—(1) CO_2_ is captured directly from the air to react with a liquid solvent (ex. water or amine-based) and form (bi)­carbonates stabilizing in the alkaline or neutral pH region, and (2) concentrated CO_2_ gas is recovered via (bi)­carbonate acidification at lower pH levels combined with a subsequent vacuum degassing. Simultaneously, the solvent’s capacity to capture more CO_2_ restores during solvent regeneration.^51^ Efficient solvent regeneration is crucial for the continuous operation of e-DAC systems, minimizing energy consumption and operational costs.^39,52^ Both CO_2_ uptake and release processes require independent R&D efforts to increase overall kinetics and thus reduce operational and equipment costs.

**Figure 1:**
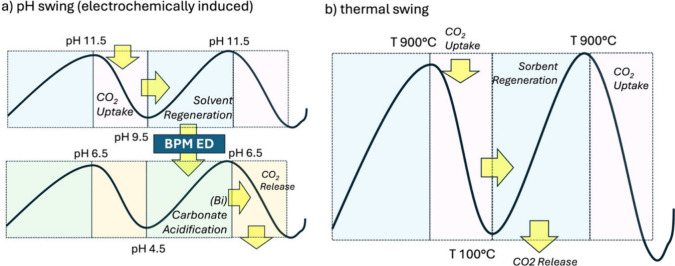
Schematic comparison of electrochemical pH-swing^16,60,61^ vs thermal-swing^25,53^ CO_2_ capture: (a) Electrochemical pH-swing process: black lines trace the solvent pH cycle—upper branch (high pH) represents CO_2_ absorption stage (as bicarbonate/carbonate), and the lower branch (low pH) represents the CO_2_ desorption stage (as dissolved CO_2_ or carbonic acid), yellow arrows indicate CO_2_ transport—either as gas entering/exiting the solvent or as dissolved (bi)­carbonate species shuttled between absorption and release zones. The key regeneration device is a bipolar-membrane electrodialysis (BPM-ED) unit, which splits water into H^+^ and OH^–^ to swing the solvent pH without external heating or pressurization. (b) Thermal-swing process: black lines show the temperature profile needed to convert carbonate solids (e.g., CaCO_3_ or MHCO_3_) into oxide or hydroxide phases and release CO_2_, then cool/reactivate for another capture cycle; yellow arrows denote CO_2_ evolution at high temperature and CO_2_ uptake upon cooling and rehydration. This conventional route relies on high-temperature calcination or pressure changes rather than electrochemical pH control.

The nonelectrochemical approaches typically employ heat^13,22,25,26,28^ or pressure changes^53^ for CO_2_ uptake and release. Most commercial CO_2_-release processes today hinge on thermal calcination—heating carbonate solids or amine-loaded sorbents^9,54–57^ to 600–900 °C to desorb CO_2_—a method widely used not only for capture, but also in cement,^58,59^ lime, and metal-oxide manufacture. This high-temperature route is energy-intensive (often burning fossil fuels), generates substantial CO_2_ and process-heat emissions, and exemplifies an industrial thermal swing by cycling between carbonate and oxide phases. In Figure 1(b), we include a schematic panel contrasting thermal calcination (carbonate ⇌ oxide + CO_2_) with the electrochemical pH swing (carbonate/bicarbonate ⇌ CO_2_ + hydroxide), underscoring their mechanistic analogy yet dramatic difference in energy form (heat vs electricity).

Both thermal/pressure and electrochemical systems can in principle operate using liquid solvents, where CO_2_-rich air is brought into contact with a solvent with capacity to extract and store CO_2_ gas molecules. The capacity of any CO_2_ capture system is dictated by the solvent’s thermodynamics (e.g., equilibrium constants for CO_2_ uptake), whereas the rate of CO_2_ capture reflects both mass transport and the intrinsic reaction kinetics between CO_2_ and the sorbent. For example, primary amines (e.g., monoethanolamine, monoEA) react rapidly with CO_2_ to form carbamate species,^44^ whereas tertiary amines (e.g., *N*,*N*-dimethylethanolamine, diMEA) typically capture CO_2_ via a base-catalyzed bicarbonate pathway—with distinct kinetic and equilibrium profiles.^62^ Secondary amines (e.g., diethanolamine, diEA) lie between these extremes,^63^ often yielding mixtures of carbamates and bicarbonates.^62,64^ Thus, the choice of primary, secondary, or tertiary amine has a profound impact on both the speciation of captured CO_2_ (carbamate: carbonate ratio) and the overall absorption/desorption kinetics.^55^

Electrochemical pH-swing platforms need not be limited to classic amine solutions; in principle, a wide array of liquid CO_2_ sorbents can be paired with membrane or electrode regeneration cycles. Strong bases such as KOH or NaOH (and promoted K_2_CO_3_ “hot potassium carbonate” formulations) could absorb CO_2_ as bicarbonate/carbonate and then be electrochemically driven back to hydroxide at ambient temperature.^65^ Ammonia-based scrubbing solutions offer milder thermal profiles and could similarly be cycled via electrochemical pH control to release CO_2_ from ammonium bicarbonate.^66^ Next-generation ionic liquids^15,67^ or deep eutectic solvents^68^—tunable for CO_2_ binding—are particularly attractive for electrochemical regeneration, since their low volatility and high ionic conductivity lend themselves to membrane-based cell designs. Bioderived amino acid salts (e.g., potassium sarcosinate) combine fast carbamate/bicarbonate chemistry with low vapor pressure and could be cycled electrochemically to toggle between capture and release.^69^ Even hybrid slurry systems—such as solvent-borne MgO/CaO or sorbent-enhanced liquids containing zeolites or MOFs—can be envisioned if their CO_2_-laden solids are redissolved or recharged by a local pH shift.^54^

Although nonaqueous solvents—particularly primary, secondary, and tertiary amines—exhibit distinct capture mechanisms (carbamate vs bicarbonate formation) and kinetic–thermodynamic trade-offs that significantly alter CO_2_ uptake rates and equilibrium loadings, an exhaustive analysis of these specific amine–CO_2_ interactions^62,70^ (or their direct interplay with membrane material chemistry) is beyond the scope of this perspective. Instead, we adopt a simplified CO_2_ capture model using alkali carbonate/bicarbonate electrolytes (e.g., KHCO_3_) to isolate key principles of the membrane-mediated pH-swing operation. The insights derived from the model are broadly applicable and could be applied in other electrochemical CO_2_ capture systems, as well. Given the shared principles such as ion transport, pH modulation, and redox-mediated CO_2_ binding, the modeling framework and results presented here provide valuable guidance for advancing multiple approaches to electrochemical carbon capture.

Electrochemical direct air capture (DAC) technologies used more specifically for CO_2_ release and solvent regeneration can broadly be grouped into three major families based on their method of inducing pH swings: membrane-driven systems,^34,36,39,71–74^ capacitive systems,^75^ and redox-mediated systems.^76,77^ Each class comes with distinct trade-offs in throughput, selectivity, operational complexity, and long-term stability, as discussed in recent reviews by Renfrew et al.,^78^ Sharifian et al.,^79^ Bui et al.,^34^ and others.^80–82^ Membrane-based pH swing systems represent a particularly promising category due to their ability to drive acid–base reactions directly by using electricity. These systems create local pH gradients through ion-selective membranes, enabling continuous and controllable CO_2_ capture and release without requiring thermal regeneration or consumable chemicals. Their architecture supports modular, stackable designs, and lends itself to integration with renewable power sources. In contrast, capacitive systems, such as capacitive deionization and its variants, operate at lower current densities and are often constrained by co-ion transport, limited selectivity, and parasitic reactions. These challenges reduce their efficiency and limit their practicality for high-throughput or long-duration DAC operation.^75,83^ Redox-mediated systems, which rely on soluble redox-active molecules to shuttle protons or modify the local pH, offer conceptual flexibility but face persistent challenges with molecular stability, slow kinetics, and very low operational current densities. These issues pose barriers to scale-up and long-term deployment.^76,77,84,85^ Meanwhile, thermal regeneration systems, including those based on amine or carbonate sorbents, are technologically mature but require external heat sources and involve materials with known degradation pathways over cycling. Their reliance on a high-temperature infrastructure introduces both economic and operational constraints, especially in an electrified energy landscape. Drawing from these comparisons, recent literature consistently points to membrane-driven electrochemical systems as the most promising platform for DAC—offering a balance of modularity, electrical efficiency, and compatibility with sustainable energy inputs.^80,86^ The next sections examine the most advanced implementation of this approach in detail.

In the following, we largely focus on the approach using BPM-ED and the closely related approaches using the AEM-EL. In Figure 2, we provide a comparison of the simplest types of design for these mentioned systems: the former BPM-ED operating in reverse bias mode, based on a two-cell configuration with repetitive sets of bipolar (BPM) and anion exchange membranes­(AEM), and the latter AEM-EL, based on repetitive membrane electrode assembly (MEA) units, each consisting of an impregnated AEM and cathode and anode electrodes with respective catalyst layers (CL).

**Figure 2:**
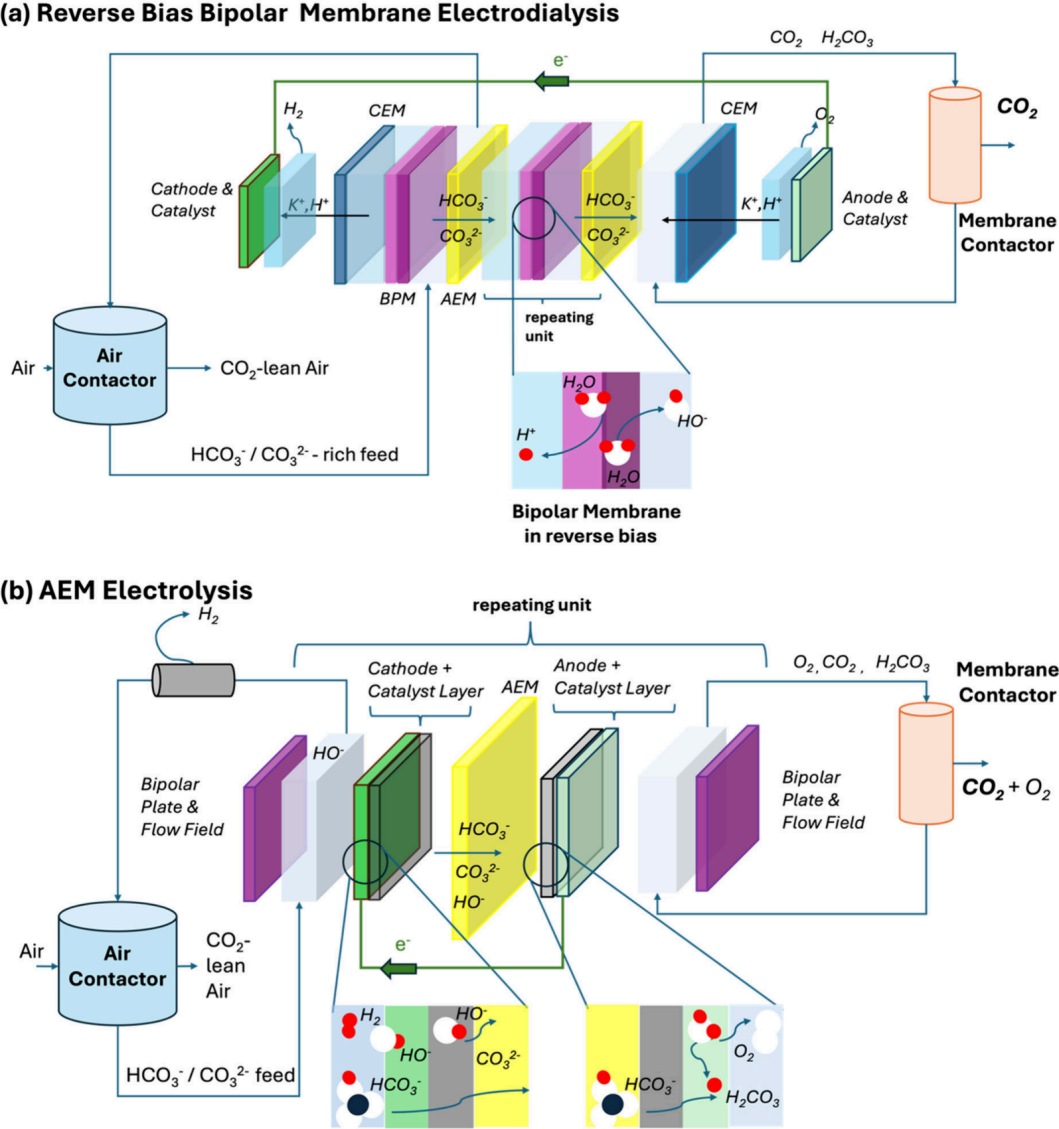
Schematic system design of two types of stacks, showing the fluxes of solvents, ions, and gaseous products involved: (a) Reverse Bias BPM-ED: A breakdown of a simple BPM/AEM-based electrodialysis stack with a cell design including 2-compartment configuration (repeating unit) including each set of BPM and AEMs, sandwiched between two cation exchange membranes (CEMs) at the interface to the electrodes (anode and cathode). Zoomed-in interface shows water dissociation (WD)—key process occurring in a BPM-ED unit under reverse bias. (b) Anion Exchange Membrane Electrolysis (AEM-EL): Flow diagram of a simplest AEM-EL process with detailed breakdown of a typical AEM electrolysis stack with membrane-electrode assembly (MEA) configuration, a cell design, each repeating unit consisting of set of cathode, anode with catalyst layers (CLs) and an AEM in-between. Zoomed-in interface shows two electrocatalytic gas evolution reactions—hydrogen gas evolution on cathode and oxygen gas evolution at the anode CL.

BPM-ED uses BPMs to dissociate water into protons and hydroxide ions under reversed bias, producing a controlled pH gradient across the membrane.^21,34,35,87^ The key BPM-ED features include (1) energy efficiency and (2) efficient ion separation. A BPM-ED stack consists of electrodes, current collectors, BPMs, and ion exchange membranes (IEMs), see Figure 2(a). A BPM itself consists of a catalyst layer (CL) sandwiched between the cation and anion exchange layers (CEL and AEL) of a specific chemical nature, with corresponding mechanical and electric properties. The reverse-bias mode refers to the operation of a BPM stack in which the electric field is applied such that the CEL face the cathode and the AEL face the anode, reversing the typical water-splitting polarity. Under this configuration, protons generated at the bipolar interface migrate toward the anode compartment while hydroxide ions migrate toward the cathode. This reversed ion flow enables selective acidification and basification across the stack, distinct from forward-bias operation, where protons move toward the cathode and hydroxide ions move toward the anode. The AEMs, as part of BPM-ED cell, as illustrated in the example in Figure 2(a), selectively allow the passage of anions. An electrodialysis (ED) unit usually comprises a single set of so-called end electrodes (end anode and end cathode) driving the electrochemical reactions with a repeating set of membranes in-between. Efficient current collectors reduce electrical resistance and ensure uniform current distribution, which is vital for optimal stack performance.^88^ Due to generally lower ranges of applied current densities, BPM-ED units produce a limited amount of H_2_ and O_2_ gas at the ED unit’s end electrodes.

The overall energy efficiency in BPM-ED is primarily defined by electricity consumption per tonne of CO_2_ removed. As discussed in detail below, the major energy losses originate from the voltage losses, due to various contributions—thermodynamic barriers for given chemical reactions, pH differences across the membranes, followed by water dissociation kinetics, cell and stack ohmic resistances, as well as solvent ohmic losses.^21,34^ Most importantly, the capacity of the membrane to transport the relevant ions with high enough selectivity while avoiding unwanted ion crossover is key for minimizing the energetic losses in the process.

The AEM-EL approaches may differ significantly in their system and stack design, the repeating cell units and components, as well as processes, costs, and operational efficiencies.^72,89–92^ The AEM-EL systems used for DAC perform dual functions; while benefiting from higher current densities (in range of 1–3 A/cm^2^) and greater CO_2_ recovery rates per unit area, they can produce significant amounts of hydrogen gas on the cathode side. H_2_ gas generation may offset some energy costs through its sale or combustion in the process. In particular, the amount of H_2_ evolved scales directly with the amount of CO_2_ captured in an AEM-EL system, whereas in a BPM system they can be decoupled.^35,79^ The system design shown in Figure 2(b) may also result in an undesirable mixing of oxygen and recovered CO_2_ at the anode side. Through smart engineering solutions or innovative process flow diagrams, the optimized system designs can prevent O_2_ and CO_2_ from mixing in the first place. Despite the larger efficiencies and higher current densities obtained in PEM electrolyzers, AEM electrolyzers are more relevant for industrial DAC because they are more cost-effective and utilize less expensive materials.^92–94^ However, AEM-EL approaches are generally not well-suited for the intermittent nature of renewable energies, as they require longer operational periods to achieve desired efficiencies and suffer from significant degradation when subjected to constant ramping up and down. Furthermore, since hydrolysis in AEM electrolyzers results in low pH values on the acidic side, ensuring chemical resistance of materials becomes crucial. Strategies such as protective coatings, acid-stable catalysts, pH buffering, or advanced electrode engineering are essential to enhance stability and performance.^37,71^

Among the various AEM-EL-based approaches, some methods focus on utilizing CO_2_ captured from DAC solvents by electrocatalytically reducing it to CO in a single-stage AEM CO_2_ electrolysis.^95–97^ These types of units offer promising pathways for CO_2_ utilization, but they also face additional inherent challenges, including thermodynamic limitations,^98,99^ resulting in extremely low energy efficiencies and low selectivity toward a single reaction product,^96,100^ all topics which are beyond the scope of this work. Nevertheless, some of the thermodynamic considerations and BPM-based strategies^100,101^ related to these systems will be discussed in later sections. Alternatively, processes aimed at pure CO_2_ release or storage, without subsequent utilization, may involve the mechanical mixing of (bi)­carbonate streams with acid to liberate the CO_2_. Remaining salt mixtures can then be regenerated into their respective acid and base counterparts using hydrolysis. The AEM-EL systems, as illustrated in Figure 2(b), include repeating units comprising liquid electrolytes separated by AEMs. Inexpensive nickel or nickel-based alloys typically catalyze for both the anode and cathode. The AEM membrane allows anionic transport (hydroxide or sulfate anions, carbonate and bicarbonate anions) while separating the gas produced at each electrode. Current collectors in alkaline systems are typically made from nickel or stainless steel, often in a mesh format to enhance contact and reduce resistance.^88,93^ Bipolar plates and MEAs play a pivotal role in ensuring efficient ion transport, gas separation, and electrical conductivity, while also providing structural support and optimizing water management for improved performance and durability.^88,95^ The design and optimization of these systems, including reducing electrode gaps, are crucial for minimizing energy losses and enhancing overall efficiency.^89^ The AEM-EL systems benefit largely from advances in AEM electrolyzers designed solely for H_2_ production.^71^

In reverse bias, for a certain low range of membrane voltages, the BPMs can operate within the limiting current density regime.^102^ The current–potential (*i–V*) curve for BPM-ED systems as shown in Figure 3(a) illustrates the relationship between the applied current density and the resulting cell voltage.^17^ At low current densities, below a certain threshold (5–10 mA/cm^2^), the *i–V* relationship is linear, and is associated with co-ion crossover^29^ and leakage.^39^ As the current density increases, the curve deviates from linearity, reflecting increased resistance and directly correlated with higher energy consumption. This deviation marks the onset of significant water splitting and other inefficiencies. Should the applied cell voltages in BPM-ED systems be too excessive, then the BPM will tend to degrade, causing a performance drop. The minimum energy consumption in lab and pilot scale BPM-ED DAC systems can be achieved within the range of 10–50 mA/cm^2^, depending on the ED stack and cell design, flow rates, pH and DIC levels.^41,72,103,104^ By optimizing the operating conditions (such as DIC and pH levels), cell design, and AEM properties for improved transport of (bi)­carbonates, can drastically improve the AEM permselectivies, improve overal BPM-ED current efficiencies, hence increase the CO_2_ recovery rates, as shown in Figure 3(b).

**Figure 3:**
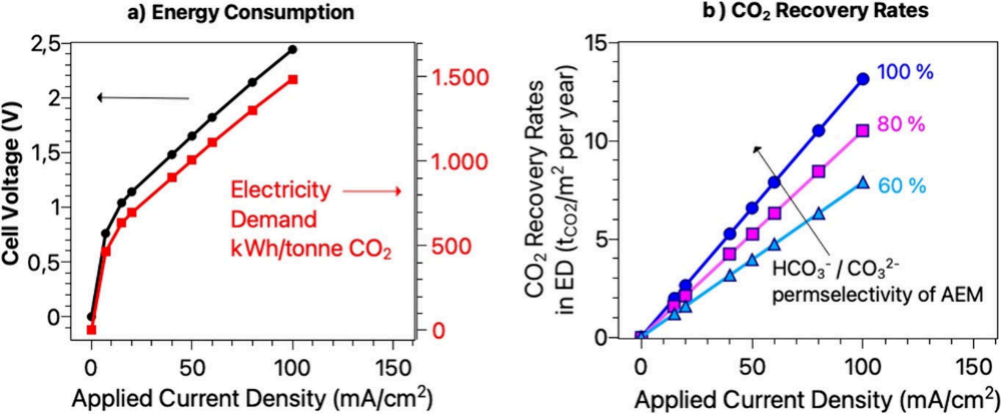
(a) Modeling of the energy consumption for a single cell in BPM-ED based CO_2_ recovery (assuming 100% AEM permselectivity toward carbonic ion species), assuming a cell voltages experimentally achieved in a cell design with a 2-compartment configuration consisting of repeating BPM/AEM sets of membranes in 1 M KHCO_3_. More details available in the datafile within the Imperial College repository^105^ https://data.hpc.imperial.ac.uk/resolve/?access=amm7-ryhz&doi=15056). Data used for modeling of cell voltages adapted from Weber and co-worker’s study (Figure 8a in ref 21). Cell voltage here is approximated by summing the BPM voltage data^21^ and the additional ohmic losses based on commercially available BPM^106^—possessing lowest resistances and aqueous solvent containing 1 M KHCO_3_ with assumed conductivity of 100 mS/cm. (b) Maximum possible CO_2_ recovery rates at various permselectivity values for carbonic species (assuming a monovalent HCO_3_^–^ ion as charge career across the AEM, *z* = 1). Sidenote: For divalent carbonate ion transfer, the regeneration rates should be divided by 2. One year of operation includes 8000 operating hours.

The voltage contributions in both BPM-ED and AEM-EL stacks depend intrinsically on the governing (electro)­chemical reactions, cell design, membrane ion transport properties, stack size, and operating process parameters, among which the most relevant are pH levels and the applied current density. In contrast to AEM-EL, the BPM-ED systems operate optimally in lower range of current densities, typically below 300 mA/cm^2^. Figure 4 illustrates the breakdown of voltage contributions in the BPM-ED and AEM-EL systems under representative operating conditions. Rather than comparing average performance across disparate literature values, our approach is rooted in first-principles analysis: we examine how current density, membrane properties, and system design influence voltage and energy consumption under the best currently demonstrated or realistically modeled conditions. To support further exploration, the underlying model and parameter set are provided in an Excel spreadsheet available in the data repository,^105^ allowing researchers to investigate the impact of altering key variables. Looking ahead, the field would benefit from more standardized benchmarking across different operational conditions to enable more consistent and comparable statistical insights. This allows us to assess which electrochemical configuration offers the most promise and where further R&D is needed to overcome specific performance bottlenecks. For instance, using the state-of-the-art data compiled by Sabatino et al.,^41^ a BPM-ED system operated at 20 mA/cm^2^ can achieve a modeled total cell voltage of approximately 1.3 V. This increases to about 1.81 V at 50 mA/cm^2^, based on combined modeling and experimental data.^21,34,36,107^ In contrast, an AEM-EL system operated at the same current density (50 mA/cm^2^) exhibits a higher voltage of approximately 1.93 V, as supported by recent experimental work^71^ and electrodialysis studies.^71,96^ These values are not intended as a direct performance ranking of fully developed systems but serve to clarify which components, such as membrane resistance, ion transport, or electrochemical overpotentials—drive differences in voltage requirements across DAC cell architectures.

**Figure 4:**
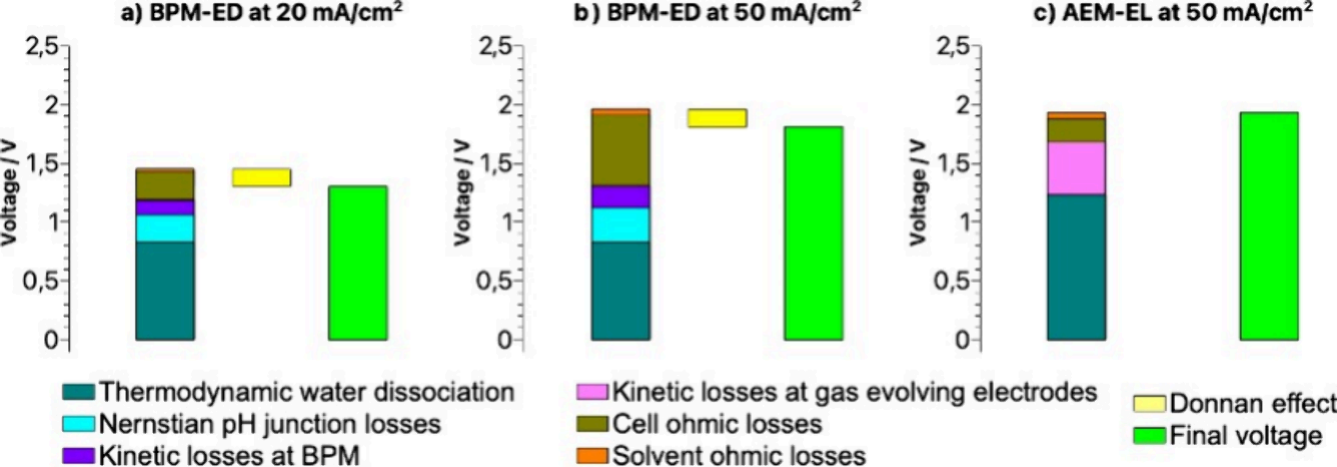
Illustrated comparison for estimated voltage contributions for various stack types at different applied current densities: (a) bipolar membrane electrodialysis (BPM-ED) at 20 mA/cm^2^ and b) BPM-ED at 50 mA/cm^2^ vs c) AEM-EL at 50 mA.cm^–2^, including: dark cyan—thermodynamic voltage losses for water dissociation (0.827 V in BPM case excluding any gas evolution reactions, and 1.229 V in AEM case including the voltage span required for the H_2_ and O_2_ gas evolution reactions); cyan—Nernstian voltage difference due to the bulk pH levels at the BPM junction (a pH difference of 4 and 5 are assumed for currents 20 and 50 mA/cm^2^ respectively); purple—Kinetic voltage losses for water dissociation at the BPM interface, data adopted from Weber and co-workers;^21^ magenta—Kinetic voltage losses related to catalytic H_2_ and O_2_ gas evolution electrode reactions per cell (BPM-ED assumes 50 cells), estimated based on Figure 5(d) from Tricker et. al;^71^ dark yellow—Voltage losses due to ohmic resistances from the cell design—BPM and AEM, as well as membrane electrode assemblies, estimated based on manufacturers/suppliers technical data sheets;^106,108^ orange—Voltage losses due to ohmic resistances of the solvent mixture, i.e., electrolyte, estimated for an aqueous solution of KHCO_3_ with concentration 1 mol/L without addition of any additives; data on ohmic resistances taken from reference;^21,106,109^ light yellow—Donnan voltage effects originating from osmotic effect across the AEM; calculation based for an aqueous solution of KHCO_3_ with concentration 1 mol/L without addition of any additives at pH 11.5 and a fixed pH of 6.5 in steady state on the acidic side of the AEM fed with deionized water with CO_2_ recovery applying vacuum on membrane degasser; light green—Overall total sum of voltages for each case. More details of the calculations are in Imperial College London repository.^105^

The intrinsic rate of the WD reaction is quantified by the turnover frequency (TOF), which varies significantly depending on whether the interface is catalyzed. In uncatalyzed BPMs, typical TOF values are low, on the order of 0.01 to 0.1 s^–1^, leading to poor performance and high junction voltages (>0.8 V). With standard catalysts such as metal oxides or functionalized organic interfaces, TOFs improve to around 1–10 s^–1^, while cutting-edge materials can push this further to 10–100+ s^–1^. These values are often estimated based on junction overpotentials and modeling assumptions due to the difficulty of measuring TOF directly.

To relate these TOF values to ion flux, we can use the product of the TOF and catalytic site density. Assuming a moderate catalyst loading of 0.01 mol active sites per m^2^ and a TOF of 10 s^–1^, the resulting ion flux is approximately 0.1 mol/m^2^/s (or 1 × 10^–5^ mol/cm^2^/s). This value aligns well with the theoretical ion flux derived from Faraday’s law at an operating current density of 20 mA/cm^2^, which yields about 0.000207 mol/m^2^/s (or 2.07 × 10^–8^ mol/cm^2^/s) for monovalent ion transport. This comparison underscores that the catalyzed BPM junction can easily sustain the required ion throughput for DAC at moderate current densities and highlights the importance of optimizing the site density and TOF to support higher loads.

Thermodynamic voltage losses are primarily dictated by the final products of the (electro)­chemical conversions (e.g., water splitting and CO_2_ chemical conversions) in each system. They are intrinsic to the reaction pathways and set the minimum voltage required to drive the process, independent of stack or system design. The minimum voltage required for water dissociation without formation of hydrogen or oxygen gases is around 0.827 V, significantly lower than value of 1.23 V in water AEM-EL that includes hydrogen and oxygen gas formation. This makes the BPM-ED approach more favorable for energy-efficient pH-swing based CO_2_ capture, as it avoids gas evolution reactions within the repeating units, see Figure 2.^35,79^ To produce hydrogen and oxygen, the AEM-EL system needs to overcome the additional kinetic overpotentials and almost a full Nernstian contribution, resulting from equalizing pH levels on both sides of the AEM at low to intermediate current densities.

Solvent ohmic losses include ionic resistances of the electrolytes on both the anode and cathode sides. Cathode electrolytes contain CO_2_ absorbing solvent, ionically conductive carbonic species dissolved in it as well as any additionally added salts for improved electrolyte properties. Cell ohmic losses include resistances brought about by the component properties and their arrangement into cells and stacks. These include electronic conductivities of the end electrodes as well as ionic conductivities and thicknesses of membranes, spacing between them suitable for optimal electrolyte flow field. BPM-ED devices aggregate larger cell ohmic resistances since the stacking includes predominantly membranes, whereas AEM-EL stacks include MEAs. As Figure 4 highlights, at higher current densities, BPM-ED exhibits significantly higher losses originating from the cell components. The current/voltage profile along the length of a BPM-ED stack may exhibit an exponential drop, which calls for additional stack segmenting, an engineering practice where auxiliary current collectors are added to circumvent the high cell ohmic losses. High applied currents cause additional concentration gradients due to ion depletion at the interfaces.

Apart from the above-mentioned nonthermodynamic losses-cell and solvent ohmic losses, the catalytic reaction activation losses also scale with the applied current density. As the applied current density increases, the nonthermodynamic losses rise because higher currents increase ion transport demands, leading to greater resistive losses and exacerbating concentration gradients. They are directly proportional to the current density, making cell and stack design optimizations crucial for energy-efficient operation at high throughput.

Figure 4 indicates how for AEM-EL, the largest voltage losses originate from the kinetic overpotentials needed for gas evolving reactions within each repeating cell of the AEM-EL stack, highlighting the necessity to speed up the electrochemical reactions by designing optimal CLs on both sides. On the other hand, in case of BPM-ED, a at given range of industrially viable applied current densitiy, the kinetic limitations for water splitting at the BPM interface remain low. Apart from above-discussed thermodynamic reasoning for this phenomenon, we also emphasize that the presence of only one set of electrodes for the entire BPM-ED stack lowers the kinetic losses related to gas evolution reactions per repeating cell unit. Furthermore, Yan et al. have proposed additional strategy to mitigate large gas evolution overpotentials at the electrodes in optimized BPM-ED units by simply looping the evolved gases. Such circulation of the electrode rinse solution ensures that the H_2_ gas generated at the cathode is oxidized at the anode, thus replacing the kinetically hindered oxygen evolution reaction, and similarly, enables supplying O_2_ from the anodic to the cathodic side, thus endorsing oxygen reduction reaction, instead of water reduction, reducing overall potential difference.^110^ BPM-ED systems mainly suffer from relatively large ohmic resistances related to the intrinsic membrane resistance and their respective ion transport properties, complex charged species interactions within CEL and AEL,^98,111–113^ as well as the geometry of the stack design, emphasizing the need for novel membrane design approaches at the molecular scale, and need for the employment of more affordable current collectors in segmenting.

The Donnan effect describes the phenomenon whereby charged membranes, such as IEMs, create an electrochemical potential difference across the membrane due to the unequal distribution of ions. This arises because fixed charges on the membrane surface (e.g., negatively charged groups in an AEM) restrict the movement of counterions, leading to an imbalance in ionic concentrations on either side of the membrane. This results in a Donnan potential, which contributes to the overall voltage in processes like electrodialysis.^114^ Donnan effect is particularly significant for BPM-ED compared to AEM-EL because BPM systems are specifically designed to sustain steep pH gradients by splitting water into protons and hydroxide ions. The fixed charges in BPM membranes amplify the Donnan effect, as the separation of H^+^ and OH^–^ creates large concentration differences at the membrane interface.^21^ This effect becomes even more critical with changing pH levels as the ionic gradients directly influence the magnitude of the Donnan potential. Furthermore, the applied current density indirectly impacts the Donnan effect because higher currents drive water splitting more intensely, exacerbating ion concentration imbalances and thereby altering the Donnan potential.^107,115^ In contrast, AEM-EL primarily operates in more uniform pH environments, where such steep ionic gradients and resulting potentials are less pronounced.

When designing, synthesizing, and manufacturing membranes for BPM-ED application in DAC, several critical factors must be considered. The optimal design of both BPM and monopolar IEMs requires a molecular-level approach to address challenges related to functionality, performance, and material durability.^38,74,116,117^ A key performance factor in BPM-ED is efficient water dissociation (WD), which is primarily facilitated at the interfacial layer of the bipolar membrane. Incorporating electrocatalysts—such as metal oxides^117,118^ or graphene oxides^107^—at this junction can enhance WD kinetics, particularly at high current densities.^36,117–120^ However, the effectiveness of the catalyst layer often influences the water availability at the interface, which is governed by the adjacent ionomer layers. Thick CEL or AEL layers can hinder water flux, leading to local dehydration and increased overpotentials. Membrane designs that reduce the CEL or AEL thickness can improve hydration but may compromise ionic conductivity or mechanical integrity, requiring a careful balance between WD performance and membrane stability. In some cases, the bipolar membrane alone may sustain sufficient water dissociation rates without the need for a catalyst, depending on the materials used and operational conditions.^38,74,87,103,107^

High ion conductivity is equally critical. Monopolar membranes (AEMs/CEMs) typically conduct H^+^/OH^–^ an order of magnitude more effectively than carbonate species, meaning that the ohmic losses across AEMs in bicarbonate form, usually ∼10–30 mS/cm, can dominate system resistance compared to the BPM junction itself. In response, several high-conductivity polymer chemistries have been developed, including quaternary ammonium-functionalized benzylated polymers and zwitterionic interlayers, some achieving >50 mS/cm in bicarbonate forms.^121^ Tailoring ion exchange site density and water uptake has further helped to reduce resistive losses and enhance ion selectivity.^122^

To mitigate ionic blockades^98,112^ and neutral species crossover in forward bias operation,^111,123^ structural modifications such as increasing fixed-charge density, introducing asymmetric AEL/CEL architectures,^107,115^ or embedding interfacial mosaics have proven effective. These configurations improve directional ion flux, reduce recombination of dissociation products, and limit parasitic transport, especially under the mixed-ion conditions typical of DAC. Examples include charge-mosaic layers, tethered proton-acceptor functionalities, and polyelectrolyte grafting techniques,^124,125^ which together enable enhanced field gradients and greater WD efficiency.

Finally, scalable manufacturing remains a practical barrier. Roll-to-roll membrane production allows for continuous, high-volume fabrication and modular customization of membrane architecture for DAC systems.^126,127^ Still, challenges persist in ensuring uniformity of layer thickness, adhesion, and catalyst integration,^128,129^ especially at high throughput. Material compatibility, drying conditions, and mechanical handling during scale-up remain key bottlenecks that must be addressed to ensure reliable industrial production of BPMs for direct air capture.

## The Bipolar Membrane Alone May Sustain Sufficient Water Dissociation Rates without the Need for a Catalyst

Beyond membrane engineering at the molecular scale and optimization at the cell and stack level, the overall CO_2_ capture performance is strongly influenced by the integrated system design. In particular, the alignment in kinetics of solvent-based CO_2_ absorption in the aeration unit and subsequent regeneration in the electrochemical pH-swing step set effectively improved the CO_2_ removal capacity. This capacity is defined by the internal flow rates and mass balances, which directly impact the required sizing of upstream and downstream units. Achieving the lowest possible energy consumption per tonne of CO_2_ removed from air depends on coordinated optimization across these system levels. Removing CO_2_ at a fixed rate using larger applied current densities can be achieved in a smaller ED stack unit with less membrane area. Moreover, an ED stack operating in reverse bias at a fixed applied current density and at a fixed flow rate stabilizes at certain steady-state DIC values, as shown in Figure 5, with CO_2_ removal capacities being directly proportional to the cross-sectional membrane area. In turn, these DIC values define the steady-state local and bulk pH values and concentrations of the respective charge carriers near membrane interfaces, directly influencing ionic conductivities, the ohmic losses, permselectivities and overall energy efficiency of the system. Therefore, when sizing the ED units, the CO_2_ absorption rate from the previous aeration step requires consideration. Even though it seems plausible that CO_2_ uptake and release rates should operate at the same levels within an integrated DAC unit, creative engineering solutions allowing optimal unit operations should be implemented to enhance the overall system performance. For example, maintaining an artificially elevated dissolved inorganic carbon (DIC) concentration in the base stream of a BPM-ED system could significantly improve conductivities, allow for higher limiting current densities (LCD), improve permselectivities and affect Donnan potentials.^130^ On the other hand, an increased ionic strength also boosts pH buffering capacity, meaning that maintaining the same pH differences across the membranes may consume more energy. In any case, the DIC concentration must not exceed the solubility product of bicarbonate at extreme operating temperatures and pressures at the higher end and not reach the depletion region at the lower end to ensure system stability and robustness. Other related system design factors, including current collector placements, larger membrane spacings, and homogeneous flow distribution, may also play a significant role in determining energy consumption.^96,131^

**Figure 5:**
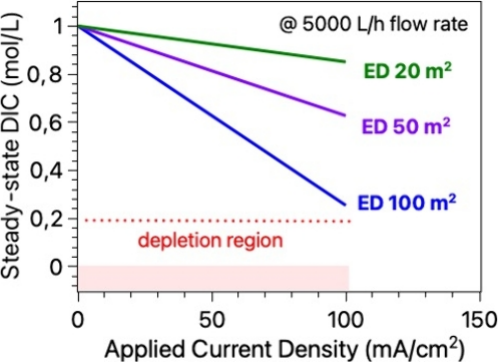
Depletion of ion concentration (dissolved inorganic carbon, DIC) during steady-state CO_2_ recovery in an ED stack with an industrially relevant liquid flow rate of 5 m^3^/h, at varied applied current densities; dotted red line represents minimum recommended operating KHCO_3_ concentration to secure low ohmic resistances across the liquid streams during ED operation; region below 0.2 mol/L DIC represents a depletion region. Three solid lines-blue, purple, and green represent steady-state DIC levels in ED stacks with (BPM, AEM) membrane areas of 100 m^2^, 50 m^2^, and 20 m^2^, respectively.

In the last section, we set out to estimate the potential to reduce the projected CO_2_ recovery and solvent renegeneration costs, thereby including progressive improvements of the electricity consumption and BPM and ED stack manufacturing costs as well as taking into consideration regular membrane replacements and auxiliary engineering expenditures related to BPM-ED operation at higher applied currents. The graph in Figure 6 presents a 20-year techno-economic outlook for a BPM-ED system integrated into a DAC plant, segmented into four 5-year intervals. In the first interval (Year 0), the graph shows only CAPEX and initial OPEX—representing the one-time capital investment required to build the system and the early operational costs. From Year 5 onward, maintenance costs are added to reflect the increasing demand for system upkeep as components such as membranes, pumps, and electrodes age. The trend of decreasing CAPEX across future build cycles illustrates the impact of the learning curve: as BPM-ED technology is deployed at larger scales and manufacturing processes improve, the capital costs of new installations are expected to decline. It is important to note that this CAPEX reduction applies to future DAC plants, not to the initial installation shown at Year 0. This structure provides a clear view of the shifting cost contributions over time and highlights the long-term economic benefits of technology maturation. Furthermore, we compare two learning curves with levelized cost of capture (LCOC) at two different process intensities: 20 vs 50 mA/cm^2^. In an example at 20 mA/cm^2^, applied current density identified to be the optimal for current state-of-the-art DAC systems,^41^ the initial CapEx vs OpEx ratio is 1:1, followed by gradually decreasing operational costs due to improved components performances and drops in renewable electricity prices, for details see Figure 6(a) caption. In Figure 6(b), pushing the applied current density toward 50 mA/cm^2^ results in higher running electricity costs per mol CO_2_ captured, however, also implies less need for the expensive bipolar membrane area, hence favorable ED unit economies, assuming permselectivities for carbonic species in this enhanced process remain equally high. Moreover, higher process intensities inevitably induce challenges during CO_2_ release, where the bubble formation can block up to 30% of the membrane area and has a potential to reduce the energy efficiencies significantly.^21^ Eisaman et al. considered additional engineering solutions, such as increasing operating pressures, to mitigate the bubble formation.^132^

**Figure 6:**
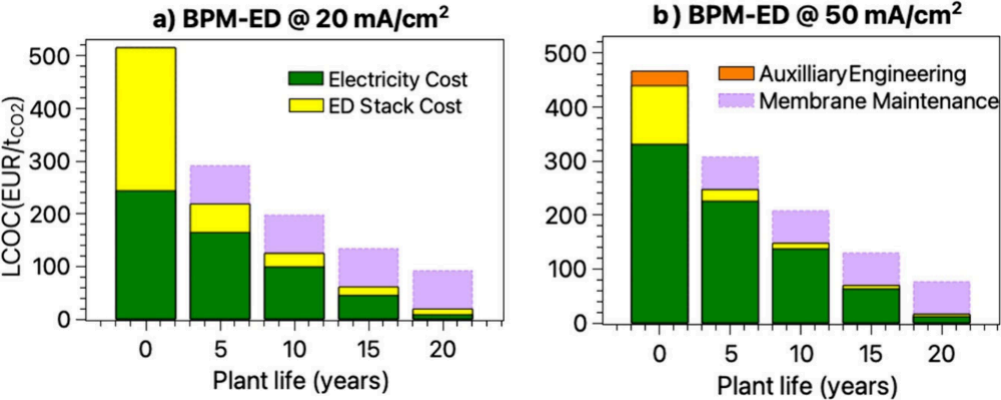
Levelized cost of capture (LCOC) for BPM-ED stage and the (technological and commercial) learning curves under two different scenarios of applied current densities: (a) 20 mA/cm^2^, and (b) 50 mA/cm^2^. Performance assumptions: Permselectivity of the AEM for carbonic species improves by 5% every 5 years, starting at 70%, ending at 90%. Cell Voltage improves by 0.05 V every 5 years, starting at 1.4 V for scenario 20 mA/cm^2^ and starting at 1.9 V, for the scenario 50 mA/cm^2^. Electricity cost assumes the use of renewable electricity with *price* dropping by 50 €/MWh every 5 years, starting at 200 €/MWh and finishing at 10 €/MWh. ED Stack Cost include all capital expenditures including cost of membranes and other accessories such as piping, regulators, manifolds, electronics and sensors*; here we assume the total cost of ED stack normalized per membrane area (in* €*/m* ^2^) decreases every 5 years by 50 €/m^2^, starting at 500 €/m^2^. Auxiliary Engineering refers to additional engineering hardware such as current collectors or high-pressure solutions to manage bubble removal. Membrane Maintenance assumes regular replacement of all membranes due to degradation or fouling at the end of their lifetime. BPM/AEM part of the cost is assumed to be 70% of initial ED Stack Cost. Frequency of change of membranes is assumed 2 times in 5 years when operating at 20 mA/cm^2^ and 4 times in 5 years when operating at 50 mA/cm^2^. Operating at 50 mA/cm^2^ includes *Auxiliary Engineering* solutions inducing additional increase in 25% of initial ED Stack Cost. Both CapEx and Membrane Maintenance costs use an annuity rate of 6% over 20 years for amortization modeling purposes.

The highest proportion of cost may arise from the regular replacement or maintenance of BPM and AEM in both cases, due to their degradation and fouling during operation under harsh conditions.^87,104,130^ At higher current densities, higher mechanical, thermal, and chemical stress on the membranes at extreme voltages, temperatures, and pH levels accelerate the degradation processes further, which requires more frequent membrane replacements. Nevertheless, in the long run, there are still tangible financial benefits for the large-scale deployment of BPM-ED based DAC plants operating at higher current densities.

Membrane lifespan is a key factor in the overall cost of BPM-ED systems, since frequent replacements can significantly increase operating expenses. Industrial BPM-ED systems target continuous operation for 5000–10000 h, yet most academic reports demonstrate stability for only hundreds to <1000 h. Sustained performance demands that membranes retain low resistance, high ion selectivity, and consistent water-dissociation activity under harsh pH gradients and high current densities.^107,117,133–135^ In long-term BPM operations, fouling remains a major challenge that limits the performance and membrane lifespan. To ensure sustained functionality, it is essential to employ both preventive and active fouling mitigation strategies. Surface modifications, such as coatings with hydrophilic polymers (e.g., polyethylene glycol, zwitterionic materials) or inorganic layers (e.g., titanium dioxide, silver nanoparticles), can reduce foulant adhesion and inhibit biofouling. Additionally, dynamic operational modes, including pulsed electric fields or reverse polarity cycling, have shown potential in dislodging deposits and minimizing scaling caused by multivalent ions. Material-level interventions also contribute to improved fouling resistance. Incorporating low-fouling ionomers or functionalized membrane layers enhances the surface properties, reducing the likelihood of accumulation.

However, fouling is not the only degradation mechanism; other critical failure modes include chemical degradation of ionomer backbones (via oxidative,^136–138^ hydrolytic, or radical pathways), mechanical fatigue due to cyclic swelling and delamination, and junction instability resulting from poor water transport or inadequate adhesion between the anion exchange layer (AEL) and cation exchange layer (CEL).^135,139^ To address these issues, current research focuses on developing chemically robust ionomers that resist hydrolysis and swelling,^140–142^ along with advanced interface engineering approaches—such as graded catalyst-layer inks and reinforced interfacial architectures—to improve interlayer cohesion. The integration of immobilized water-dissociation catalysts helps sustain activity over prolonged periods. At the device level, strategies like periodic polarity reversal for in situ cleaning and embedded impedance monitoring for predictive maintenance are being explored to further extend the membrane lifespan and reliability.

Ultimately, optimizing both material properties and operating conditions is critical to minimizing degradation and fouling. Flow Accelerated stress testing and computational modeling can help identify optimal membrane chemistries, charge carriers, pH environments, ionic strengths, and mechanical designs for flow fields and spacers. Engineering BPMs with high permselectivity for target ions and tailored residence times can reduce voltage losses, while enhancing durability. A multiscale understanding of BPM chemistry, structure, and transport phenomena, followed by scalable manufacturing, will be key to achieving long-term stability under extreme operating conditions.

Similar cost estimates made for the AEM-EL system, as those made for BPM-ED in Figure 6, would result in even higher initial operational costs due to increased electricity consumption and on average higher electricity prices, as these systems inherently operate at higher currents, higher voltages, and exhibit significant limitations to benefit from intermittent but affordable renewable electricity. On the other hand, their significantly larger operating current densities—up to 10 times higher than BPM-ED systems—result in lower initial capital expenditures (CapEx) and reduced maintenance costs over time. However, to fully assess the viability of such systems, further modeling, calculations and pilot testings are necessary. They must account for hydrogen management and storage systems to design and optimize the process while benchmarking its competitiveness against existing hydrogen electrolysis and DAC technologies.

## Even though It Seems Plausible That CO_2_ Uptake and Release Rates Should Operate at the Same Levels within an Integrated DAC Unit, Creative Engineering Solutions Allowing Optimal Unit Operations Should Be Implemented to Enhance the Overall System Performance

So far, experimentally validated BPM-ED systems for bicarbonate-based CO_2_ regeneration have demonstrated operation within a moderate current density window. Foundational lab-scale studies using **Neosepta BP-1** and *Fumasep FBM* ^106^ membranes have shown performance up to 10–100 mA/cm^2^, with specific energy consumptions ranging from 630 to 1890 kWh per tonne CO_2_ depending on system integration and solvent selection^39,46,51^ More recent efforts with improved interfacial engineering and thin-layer architectures^101^ have reached stable operation near 300 mA/cm^2^; nonetheless, such high-current systems have yet to be validated with full carbonate/bicarbonate transport in DAC-relevant media.

AEM electrolysis platforms, particularly those adapted from alkaline water electrolysis, show stronger maturity and scalability. State-of-the-art systems using poly­(aryl piperidinium)^136^ and low-cost FAA/Fumasep-type AEMs^109^ operate up from 1 to 2 A/cm^2^, with peak reported densities as high as 7.7 A/cm^2^ in optimized cells.^109,136,143^ While energy consumption for DAC-specific regeneration is often not explicitly measured, these platforms provide a strong technological base for future anodic CO_2_ release applications, especially where integration with amine or bicarbonate solvent loops is planned.

From Table 1, we observe that BPM-ED development remains constrained by two main bottlenecks: (i) insufficient ion-selectivity and conductivity for carbonate/bicarbonate transport, which results in high overpotentials even at moderate current densities, and (ii) a lack of long-term performance validation under realistic DAC cycling and solvent regeneration conditions. Vallejo Castaño et al.[Bibr ref51] (2024) demonstrated operation at 50 mA/cm^2^ with the lowest reported energy use of ∼2440 kWh/tonne CO_2_, however, this still exceeds the 1500 kWh/tonne threshold often cited as a benchmark for competitive DAC. Conversely, AEM electrolysis systems exhibit stronger maturity and operational headroom, with demonstrated current densities of 100–2000 mA/cm^2^ in DAC-adapted setups^136^ and peak values up to 7.7 A/cm^2^ in advanced water electrolyzers.^143^ However, they are limited by the chemical durability of the AEMs, especially under high pH and oxidative potentials, which are typical in CO_2_ solvent regeneration environments. Looking forward, BPM-ED systems operating within a 50–200 mA/cm^2^ window could potentially reach sub-1500 kWh/tonne CO_2_ energy intensities, provided cell voltages remain below 3 V and membranes are redesigned for efficient carbonate transport and chemical durability. For AEM-EL, the immediate opportunity lies in scaling alkaline electrolysis stacks toward DAC use cases, leveraging their existing industrial deployment base while addressing membrane oxidation and compatibility with carbonate-containing feeds.

**Table 1:** Comparison of State-of-the-Art Lab Scale Experimental Achievements Using BPM-ED and AEM-EL for DAC Relevant Current Ranges and Systems

Ref.	Study/Reference	System Type	Membrane Type	Current Density (mA/cm^2^)	Energy (kWh/tonne CO_2_)	Notable Features
^39^	Eisaman et al. (2011)	BPM-ED	Neosepta BP-1 (BPM), AMX (AEM), CMX (CEM)	∼10–100	630–1260	First DAC-relevant BPM-ED study; recovery from bicarbonate
^46^	Eisaman et al. (2012)	BPM-ED (Seawater)	Neosepta BP-1, AMX/CMX	∼10–100	630–1890	BPM-ED adapted for CO_2_ separation from seawater
^51^	Vallejo Castaño et al. (2024)	BPM-ED	Fumasep FBM	4–100	2444 at 50 mA/cm^2^	Solvent regeneration via BPM-ED; stack energy evaluated
^108^	Blommaert et al. (2021)	BPM-ED	Fumasep FBM	25	Not reported	Orientation effects of BPM in CO_2_-rich MEA systems
^101^	Petrov et al. (2024)	BPM Electrolysis	Custom asymmetric BPMs (polymeric junction)	Up to 300	Not specified for DAC	Stability and high-current BPM operation; stack-relevant
^143^	Chen et al. (2021)	AEM Electrolysis	Poly(aryl piperidinium)	Up to 7680	Not CO_2_-specific	Very high current density; DAC relevance inferred from material development
^109^	Vincent et al. (2021)	AEM Electrolysis	Low-cost AEMs (FAA or Fumasep, unspecified)	Up to 1000	Not specified	Focus on impedance and cost-effective membrane setups
^136^	Lim et al. (2024)	AEM Electrolysis	Piperidinium-based AEMs	Up to 2000	Not DAC-specific	Studies oxidative degradation in DAC-relevant current range

Electrochemistry based DAC processes will play a crucial role in the sustainable energy landscape. The pH-swing approach is an efficient engineering method to reduce CO_2_ from the air, leveraging pH changes to facilitate CO_2_ capture and release, potentially reducing overall energy consumption and improving scalability.^21,34,39,79^ With advancements, these methods can become cost-competitive, particularly as the technology matures and economies of scale are realized. The BPM-ED concept is currently one of the most promising and most-reported electrochemical approaches for effective CO_2_ recovery and solvent regeneration. To drive down CO_2_ capture costs, the focus must be on minimizing CapEx and maintenance through smart design while ensuring seamless integration with intermittent renewables to keep OpEx low.

## To Drive down CO_2_ Capture Costs, the Focus Must Be on Minimizing CapEx and Maintenance through Smart Design, while Ensuring Seamless Integration with Intermittent Renewables to Keep OpEx Low

In Figure 7, we illustrate the need for synergetic scientific research and engineering development efforts at various scales, from DAC systems at plant level, toward BPM-ED at a stack microlevel, down to development and manufacturing of more durable and affordable membrane materials at the molecular scale. Moderate range of current densities, combined with well-built ED stacks with optimal size, well-designed cell and system configurations, and high-quality membrane materials, is essential for minimizing energy consumption and enhancing the overall efficiency of BPM-ED based CO_2_ capture systems.

**Figure 7:**
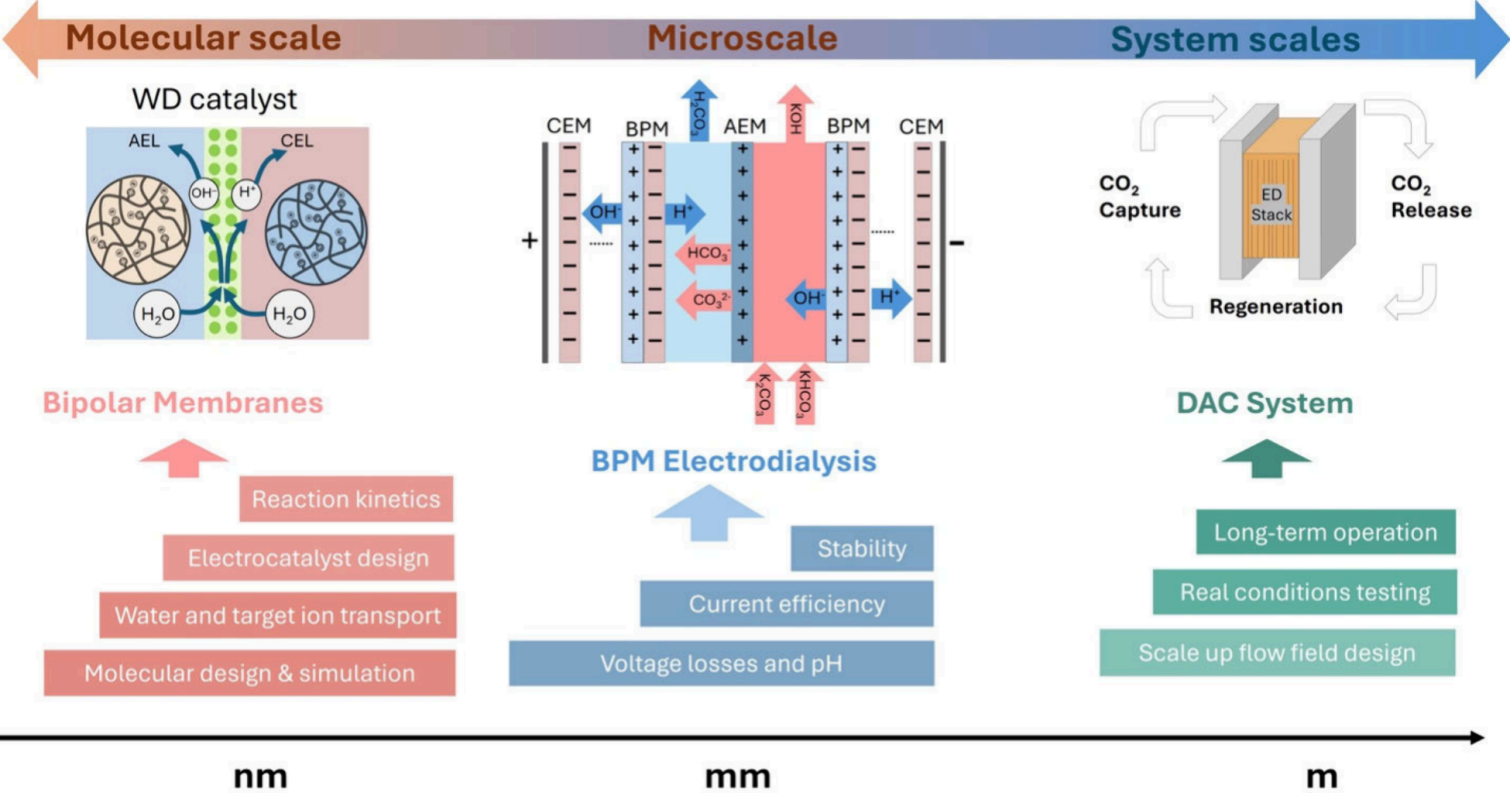
Challenges to address at various scales: (a) molecular—novel polymer materials discovery, synthesis and functionality at molecular scale (illustrations on the left), (b) microscale—cell and ED stack design for improved ohmic resistances (illustration in the middle), and (c) DAC system scale, fully scaled-up ED system design tested under realistic operating conditions for long-term. Parts of this figure (WD catalyst part) were reproduced with permission from ref 118. Copyright 2024 Springer Nature, Ltd.

The key engineering and scientific challenges to address at the *system-*, *micro-* and *molecular* scale are summarized below:

To accelerate the industrialization of BPM-ED DAC systems, system-level research should focus on the coordinated optimization of unit operations and dynamic process integration. As illustrated on the far right-hand side of Figure 7:

(1) First, efforts should be made to decouple CO_2_ uptake, solvent regeneration, and degassing steps, thereby relaxing the tight design dependency between membrane area and capture rate. This decoupling would allow each unit operation to run at its optimal performance condition, improving cost-effectiveness and flexibility.
(2) Second, maintaining elevated concentrations of dissolved inorganic carbon (DIC) within the base stream of the BPM-ED system—while staying within safe solubility limits—can significantly improve conductivity and allow operation at higher limiting current densities (LCD). This strategy enhances ion transport, improves permselectivity, and reduces resistive losses, though care must be taken to avoid bicarbonate precipitation or DIC depletion at operating extremes.
(3) Third, system designs must incorporate mechanisms to manage voltage fluctuations caused by intermittent renewable energy sources. This can be achieved by integrating energy storage or buffering technologies that stabilize current loads, ensuring consistent ion transport and minimizing voltage inefficiencies.

At the *micro*scale, as shown in the center of Figure 7, the design and optimization of -ED systems must align with

(1) Advancing BPM-ED performance at the microscale requires fine-tuning operating conditions, particularly solvent flow rates, pH, ionic strength, and limiting current density (LCD). These parameters must be optimized in concert with membrane selection, cell geometry, and stack design to enhance ion transport efficiency and minimize energy losses.
(2) Special attention should be given to managing local pH environments, as (bi)­carbonate species significantly affect conductivity and electrochemical behavior. Strategies to reduce boundary layer thickness and increase LCD will be essential for improving throughput without compromising selectivity.
(3) Leveraging osmotic concentration gradients and Donnan potential effects at membrane interfaces presents a promising, underexplored design pathway. In particular, the use of deionized water at the anode side—rather than conventional salt addition—can intentionally create beneficial electrochemical gradients that enhance proton transport and water dissociation at the BPM interface.
(4) R&D efforts should target optimal pH differentials (in the range of 4–5 units), which have been shown to balance energy efficiency with ion selectivity while minimizing voltage losses from steep Nernstian gradients.
(5) Microscale designs must anticipate industrial-scale demands: achieving high ionic flux at low energy input, maintaining concentration and pH gradients within stable limits, and integrating auxiliary elements that manage pressure, gas evolution, and long-term durability. Such optimizations lay the foundation for reliable, scalable BPM-ED DAC operation under real-world conditions.
(6) Lastly, further gains can be achieved by engineering the stack architecture itself. Optimization of current collector configurations, membrane spacing, and flow distribution can reduce ohmic losses and parasitic power demand. These system-level strategies, when implemented in parallel, can significantly improve the techno-economic viability and operational robustness of BPM-ED DAC technologies.^86^

## Leveraging Osmotic Concentration Gradients and Donnan Potential Effects at Membrane Interfaces Presents a Promising, Underexplored Design Pathway

At the *molecular* scale, as depicted on the far-left side of Figure 7, future research should focus on the strategic design of membrane materials that improve performance without compromising durability or manufacturability.

(1) Development of chemically stable ionomers, particularly for anion exchange layers (AELs), remains essential for enabling sustained bicarbonate and carbonate transport under high pH and electric field conditions typical in BPM-ED DAC. Ether-free, alkaline-stable polymers such as poly­(arylene piperidinium) offer promising pathways and should be further optimized for mechanical and chemical compatibility between AEL and CEL layers.
(2) Water dissociation remains a key performance bottleneck. While incorporation of catalytic interfacial layers (CLs) such as metal oxides (e.g., TiO_2_, SnO_2_, GrOx) can reduce kinetic overpotentials,^36,117,119,137^ their use must be balanced against increased mass transport resistance and potential water starvation. In some applications, the bipolar membrane alone may be sufficient to achieve effective water dissociation, eliminating the need for added catalyst layers. Material-specific assessment is required to determine the optimal strategy. Overpotential losses at the water dissociation (WD) interface may be modest at moderate current densities, whereas interfacial kinetics, membrane architecture, and ion transport limitations are dominant bottlenecks highlighting the need for holistic BPM optimization beyond just the catalyst layer. At higher operating intensities, the kinetic losses become more significant, justifying higher catalyst loadings in the CL to sustain WD.
(3) Improving the conductivity of monopolar membranes for K^+^/(bi)­carbonate transport is another priority. Conventional AEMs exhibit 3–10-fold lower conductivity than proton-conducting membranes, creating ohmic losses that exceed the BPM interface resistance. High-conductivity polymers and ionomer engineering—such as increasing fixed-charge density, introducing asymmetric membrane architectures, or embedding zwitterionic interlayers—can alleviate this bottleneck by minimizing co-ion crossover and ionic blockade.
(4) Roll-to-roll manufacturing must be further developed as a scalable, reliable approach for BPM production. Key efforts should focus on achieving uniform layer thickness, strong interfacial adhesion, and defect-free integration of interfacial or catalyst layers, while ensuring compatibility with DAC operating environments. Success in scaling production while preserving performance will be critical to enabling broad industrial application of BPM-ED DAC systems.

In parallel, a deeper mechanistic understanding of degradation pathways will inform the design of more durable BPMs, such as through the addition of protective surface coatings.^88,144^ Reducing BPM production costs and extending operational lifetimes are crucial to improving the cost-effectiveness of BPM-based systems in applications such as electrochemical DAC. Alternatively, membrane-free electrolytic architectures are also being investigated as a means of eliminating membrane replacement costs entirely.^145^

Additionally, to overcome removal intensity limitations originating from the CO_2_ absorption part of the process, improving electrolyte/solvent formulations for better CO_2_ uptake kinetics and achieving higher dissolved inorganic carbon (DIC) concentrations beyond aqueous equilibrium are critical. This could involve modifying solvent chemistry through additives, employing catalysts (homogeneous or enzymatic),^78^ or integrating materials in the liquid electrolytes that absorb CO_2_, (known as porous liquids),^146^ such as metal–organic frameworks^147^ (MOFs) for enhanced CO_2_ uptake.^78^

In summary, cost projections reveal comparable total expenditures for both low- and high-intensity DAC operations, corresponding to current densities of 20 and 50 mA/cm^2^, respectively. Lower current density systems entail reduced operational expenditures (OpEx) but demand significantly higher upfront capital investments (CapEx). In contrast, processes operating at higher current densities offer lower initial CapEx but incur elevated OpEx, particularly due to more frequent maintenance requirements. In the near term, low-to-moderate current densities are technologically feasible with commercially available components, though they remain far from optimal energy efficiency. Advancing R&D in system scaling, stack integration, and hybridization with intermittent renewable sources will be critical to optimizing electrodialysis (ED) stack designs and electrochemical DAC configurations. These efforts will also support smoother integration with downstream CO_2_ utilization technologies, enhancing the overall system flexibility and deployability. Over time, such developments will accelerate learning rates and enable the successful deployment of intensified DAC processes operating at higher current densities. Parallel laboratory-scale research into novel membrane materials, interface engineering, and synthesis methods must continue—accompanied by rigorous testing and benchmarking at larger ED and DAC system levels—to ensure long-term performance viability. Progress in the design and scalable manufacturing of advanced anion-, cation-, and bipolar membranes, along with improved catalyst layers for efficient water dissociation and optimized solvent formulations for electrochemical pH swing systems, will be key drivers of DAC intensification. These integrated innovations will ultimately make broad electrification of CO_2_ capture a technically and economically competitive climate solution.
